# Enhancement of Fluoride’s Antibacterial and Antibiofilm Effects against Oral *Staphylococcus aureus* by the Urea Derivative BPU

**DOI:** 10.3390/antibiotics13100930

**Published:** 2024-09-30

**Authors:** Jia Liu, Qingqing Weng, Dongxin Da, Shuran Yao, Ying Zhang, Yang Wu

**Affiliations:** 1 Department of Preventive Dentistry, Shanghai Stomatological Hospital, School of Stomatology, Fudan University, Shanghai 200120, China; liujia0699@163.com (J.L.); wengqq2017@163.com (Q.W.); orientalstarddx@163.com (D.D.); yjykqyx@163.com (S.Y.); 2 Shanghai Key Laboratory of Craniomaxillofacial Development and Diseases, Fudan University, Shanghai 200120, China; 3 Key Laboratory of Medical Molecular Virology of Ministries of Education and Health, School of Basic Medical Science, Institutes of Biomedical Sciences, Shanghai Medical College, Fudan University, Shanghai 200032, China

**Keywords:** fluoride, *Staphylococcus aureus*, BPU, biofilm, oral infections, synergistic antibacterial effect, fluoride ion channels

## Abstract

**Background:** The oral cavity is an important but often overlooked reservoir for *Staphylococcus aureus*. The effective control and prevention of *S. aureus* colonization and infection in the oral and maxillofacial regions are crucial for public health. Fluoride is widely used in dental care for its remineralization and antibacterial properties. However, its effectiveness against *S. aureus* has not been thoroughly investigated. **Objectives:** This study aimed to evaluate the potential of combining sodium fluoride (NaF) with compounds to enhance its antibacterial and antibiofilm effects against *S. aureus*. **Method:** We found that a urea derivative significantly enhances the efficacy of fluoride by promoting the retention of fluoride ions within the cells. The synergistic antibacterial and antibiofilm effects of BPU with NaF were confirmed through various assays, including checkerboard assays, time-kill assays, and growth curve analysis. These findings were further supported by additional methods, including transmission electron microscopy (TEM), in silico simulations, and gene overexpression studies. **Results:** These findings suggest that targeting fluoride ion membrane exporters could enhance antibacterial efficacy. When combined with fluoride, 1,3-Bis [3,5-bis(trifluoromethyl)phenyl]urea (BPU) showed increased effectiveness in inhibiting *S. aureus* growth and reducing established biofilms. **Conclusions:** This novel combination represents a promising therapeutic strategy for treating biofilm-associated *S. aureus* infections, offering a new strategy in oral healthcare. To fully evaluate the clinical potential of this synergistic therapy, further in vivo studies are essential.

## 1. Introduction

*S. aureus* poses a significant public health threat due to its ability to colonize, proliferate, and invade various body regions, leading to severe morbidity and mortality [1]. The oral cavity, particularly when colonized by antibiotic-resistant strains of *S. aureus*, can serve as a critical reservoir for infection transmission. This can facilitate the spread of bacteria to distant sites, including the bloodstream or lungs, an often overlooked aspect [2,3]. Methicillin-resistant *S. aureus* (MRSA) is of particular concern, as it remains a leading cause of both healthcare-associated and community-acquired infections globally. The widespread prevalence and severity of MRSA infections significantly contribute to global healthcare costs. This is why the World Health Organization (WHO) has designated it as a priority pathogen [4]. Given the increasing concern regarding antibiotic resistance, this study explores a novel combination therapy using BPU and fluoride, aiming to enhance the antibacterial and antibiofilm effects against *S. aureus*, including resistant strains.

*S. aureus* has been detected in various oral sites, including the oral mucosa, dental plaque, periodontal pockets, the dorsal surface of the tongue, saliva, denture surfaces, and areas affected by angular cheilitis [5,6,7,8]. *S. aureus* is also linked to several oral conditions and infections, including maxillofacial space infections, angular cheilitis, oral mucosal diseases, dry socket, parotitis, jaw osteomyelitis, and peri-implantitis [9,10,11,12,13,14]. Additionally, *S. aureus* has been implicated in the challenges of treating periodontal disease and oral cancers [15]. Given the increasing antibiotic resistance, including in oral infections, addressing *S. aureus* with novel therapeutic approaches is crucial for improving outcomes in treating periodontal disease and oral cancers.

*S. aureus* biofilms are complex bacterial communities encased in a matrix that enhances antibiotic resistance and evades immune responses, resulting in more persistent and difficult-to-eradicate infections [14]. The oral environment provides ideal conditions for *S. aureus* biofilm formation, including abundant nutrients, optimal temperature, and moisture [16]. Managing *S. aureus* biofilms in the oral cavity poses significant challenges. Standard antibiotic treatments are often insufficient in eradicating *S. aureus* biofilms. Future research should prioritize developing more effective strategies to combat biofilms and improve therapies targeting biofilm-associated infections.

An oral chlorhexidine (CHX) rinse is effective in controlling oral bacteria, but it is associated with significant side effects and limitations. Severe allergic reactions, including hives, rashes, difficulty breathing, and swelling of the face or throat, can occur. Long-term use may also cause swollen salivary glands, mouth irritation, tooth staining, and altered taste, which can negatively impact patient compliance. Additionally, there is growing concern that CHX use may contribute to the development of antibiotic-resistant bacterial strains. These challenges underscore the urgent need for new strategies to address these limitations and improve treatment outcomes.

Fluoride has been extensively used in dental care worldwide for more than half a century. It protects enamel and dentin by promoting mineralization and remineralization. Fluoride also inhibits key bacterial enzymes, such as enolase and F-ATPase, thereby disrupting bacterial growth and metabolism [17]. Higher fluoride concentrations enhance its antibacterial efficacy against *S. aureus* [18]. Excessive intake of fluoride poses significant risks, such as dental fluorosis and skeletal fluorosis [19]. Thus, it is crucial to develop innovative approaches to enhance fluoride’s antibacterial efficacy against *S. aureus* in the oral cavity while minimizing potential adverse effects.

**Figure 1:**
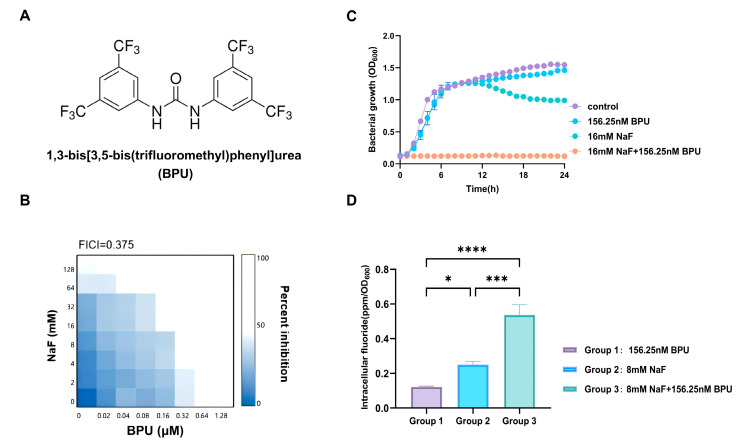
Synergistic antibacterial activity of BPU and fluoride against *S. aureus.* (**A**) Chemical structure of the BPU molecule. (**B**) The checkerboard assay showing the interaction between BPU and fluoride. (**C**) Growth curves of *S. aureus* in different TSB media were monitored by measuring the optical density at 600 nm (OD600) every 1 h. (**D**) Intracellular fluoride levels were measured using ion chromatography (n = 3, *: *p* < 0.05, ***: *p* < 0.001, ****: *p* < 0.0001).

Extensive experiments were conducted to screen both reported and novel compounds. We identified a compound that synergizes with fluoride, enhancing its antibacterial and antibiofilm efficacy against *S. aureus*. This compound, BPU (Figure 1A), was selected for further investigation due to its unique chemical structure, which suggests strong interactions with bacterial targets when combined with fluoride [5]. Previous studies have indicated that BPU may enhance the antibacterial activity of fluoride against other bacterial species, making it a promising candidate for combating *S. aureus* in the oral environment [20,21]. However, previous studies did not address the synergistic inhibition or antibiofilm activity of BPU and fluoride against *S. aureus.* To date, the specific molecular target of BPU remains unclear. We also tested other available compounds, but they did not show significant synergistic inhibition of *S. aureus* when combined with fluoride. Nevertheless, this approach could offer a cost-effective alternative to developing new compounds.

Previous research has established that fluoride ion channels play a crucial role in bacterial fluoride resistance, with the *crcB* gene being a key example in several bacterial species [22]. We identified two adjacent genes in *S. aureus* USA300 that may be involved in fluoride ion transport: *crcB* (Gene ID: USA300HOU_RS09465), located at positions 1,909,752–1,910,117 on the genome, and USA300HOU_RS09470, located at positions 1,910,114–1,910,467. These genes overlap by four bases, indicating a potential cooperative role in facilitating fluoride efflux. For simplicity, we refer to these genes as *crcB*1 and *crcB*2. In other bacteria, the *crcB* gene is known to facilitate fluoride efflux by maintaining intracellular fluoride levels below inhibitory concentrations, thereby reducing fluoride toxicity [23]. Although the regulatory mechanisms of these genes in *S. aureus* remain unknown, their proximity and potential role in fluoride resistance make them compelling candidates for further investigation. We explored the possibility that these genes could serve as molecular targets for BPU, a compound that enhances fluoride’s antibacterial activity, providing a novel approach to improving its efficacy against *S. aureus* in the oral cavity.

This study aimed to (1) investigate the antibacterial and antibiofilm efficacy of BPU combined with fluoride against *S. aureus*; (2) explore BPU’s molecular targets in *S. aureus*; and (3) assess BPU’s cytotoxicity on human gingival fibroblasts (HGFs) to evaluate its clinical suitability. Checkerboard assays, growth curves, and ion chromatography were used to verify the synergistic antibacterial effects. Confocal microscopy, TEM, and crystal violet staining were employed to assess antibiofilm activity. Homology modeling, molecular docking, and gene overexpression mutants were used to identify potential molecular targets. Finally, the safety of BPU on HGFs was confirmed, supporting its potential for clinical application.

## 2. Results

### 2.1. Synergistic Activity of BPU with Fluoride

To assess the efficacy of NaF in inhibiting *S. aureus*, we determined the minimum inhibitory concentration (MIC) values of NaF for several *S. aureus* strains, including USA300, USA500, ATCC 29213, Newman, and a clinical isolate. The MIC values for all strains were consistently 128 mM. To identify potential compounds that could synergistically enhance fluoride’s antimicrobial activity, we performed checkerboard broth microdilution assays, testing NaF in combination with various compounds. BPU (Figure 1A) exhibited a synergistic interaction with NaF in controlling *S. aureus*, with a fractional inhibitory concentration index (FICI) of 0.375 (Figure 1B). Notably, the MIC of fluoride decreased eight-fold when 156.25 nM BPU was added (Table 1). Growth curves further confirmed the significant inhibition of *S. aureus* by the combination of 156.25 nM BPU and 16 mM NaF in a tryptic soy broth (TSB) medium (Figure 1C). Additionally, ion chromatography assays demonstrated a significant increase in intracellular fluoride levels when BPU and NaF were used together, compared to either agent alone (Figure 1D).

As shown in Table 1, Compound **1** (BPU) exhibited the lowest FICI, indicating the strongest synergy. Other compounds tested, such as Compound **5**, showed irrelevance in their interaction with NaF, as indicated by FICI values > 0.5. This suggests that BPU is a more effective enhancer of fluoride’s antimicrobial activity compared to other structurally similar compounds.

### 2.2. Time-Kill Assay and Ultrastructural Alterations of S. aureus with Different Treatments

**Figure 2:**
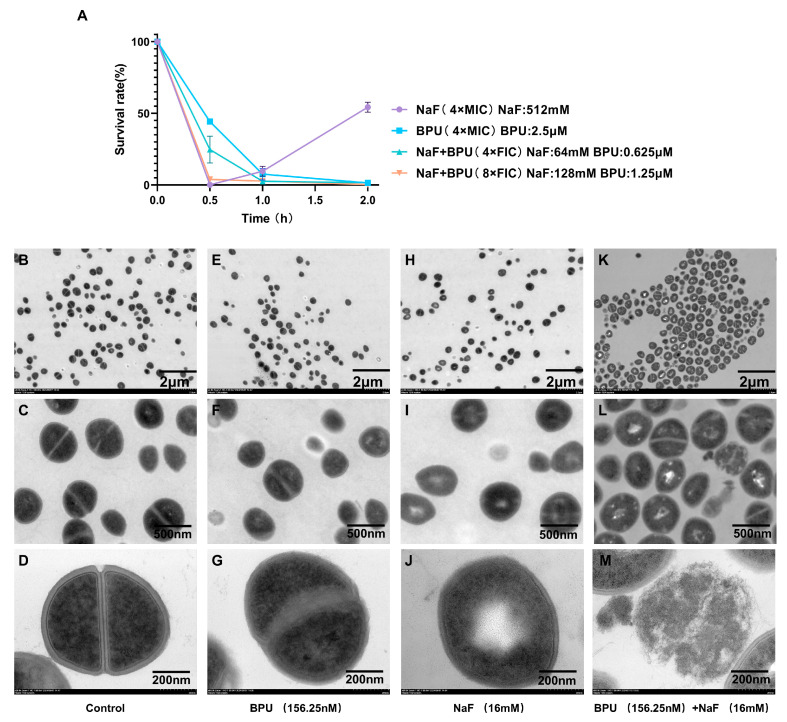
(**A**) Time-kill assay of *S. aureus* under different treatments, presented as survival rate. Data are expressed as mean ± SEM, n = 3. (**B**–**M**) TEM images of *S. aureus* under different treatments. (**B**–**D**) Control group: intact cell walls and well-defined structures. (**E**–**G**) BPU (156.25 mM) treatment: slight deformation, irregular cell walls, and minor cytoplasmic changes. (**H**–**J**) NaF (16 mM) treatment: pronounced disruption, including vacuoles in the cytoplasm. (**K**–**M**) Combined BPU and NaF treatment: severe alterations, including extensive cell wall disruption, vacuole formation, and cytoplasmic leakage. Images are presented at different magnifications: (**B**,**E**,**H**,**K**) 4000× (×4.0 k) with a scale bar of 2 µm; (**C**,**F**,**I**,**L**) 24,000× (×24.0 k) with a scale bar of 500 nm; (**D**,**G**,**J**,**M**) 60,000× (×60.0 k) with a scale bar of 200 nm.

A time-kill assay was conducted using *S. aureus* USA300 to investigate the synergistic bactericidal activity of BPU with fluoride. Various treatments were applied, including NaF alone at 4 × MIC (512 mM), BPU alone at 4 × MIC (2.5 µM), and combinations of 4 × FIC (NaF: 64 mM, BPU: 0.625 µM) and 8 × FIC (NaF: 128 mM, BPU: 1.25 µM). The results (Figure 2A) showed that NaF alone (512 mM) reduced bacterial survival to nearly 0% within 0.5 h; however, survival increased to approximately 30% by 2 h, indicating partial regrowth. BPU alone (2.5 µM) reduced bacterial survival to around 5% within 1 h, with no significant recovery observed thereafter. In contrast, the combination treatments exhibited significantly enhanced bactericidal activity compared to either agent alone. The 4 × FIC combination (NaF: 64 mM, BPU: 0.625 µM) reduced bacterial survival to about 20% at 0.5 h and further decreased it to around 5% by 1 h, with no subsequent regrowth. The 8 × FIC combination (NaF: 128 mM, BPU: 1.25 µM) reduced bacterial survival to approximately 5% within 0.5 h, and this level was maintained without regrowth throughout the 2 h observation period.

TEM analysis was performed to observe the ultrastructural alterations in *S. aureus* under different treatments: control, BPU (156.25 mM), NaF (16 mM), and a combination of BPU (156.25 mM) and NaF (16 mM). In the control group (B, C, and D), over 95% of the cells exhibited intact, smooth cell walls and well-defined cellular structures with no visible damage. BPU treatment (156.25 mM) (E, F, and G) caused slight deformation, with approximately 20% of cells showing cell wall irregularities and minor cytoplasmic changes. NaF treatment (16 mM) (H, I, and J) caused more pronounced disruption in around 40% of cells, including less distinct cell wall boundaries and vacuoles in the cytoplasm, indicating structural damage. The combination treatment (BPU: 156.25 mM and NaF: 16 mM) (K, L, and M) caused severe cellular alterations in over 80% of cells. TEM images showed extensive disruption of the cell wall, with sections of the membrane visibly detached from the bacterial surface. In addition, 40–50% of the cells contained vacuole-like structures within the cytoplasm (K and L). Cytoplasmic content leaked through compromised membranes, leading to extensive membrane rupture (M). These findings illustrate significant structural damage, particularly in the combined treatment group, where the most severe disintegration of cell walls and leakage of cellular contents occurred.

### 2.3. Effects of BPU on Different S. aureus Strains

**Figure 3:**
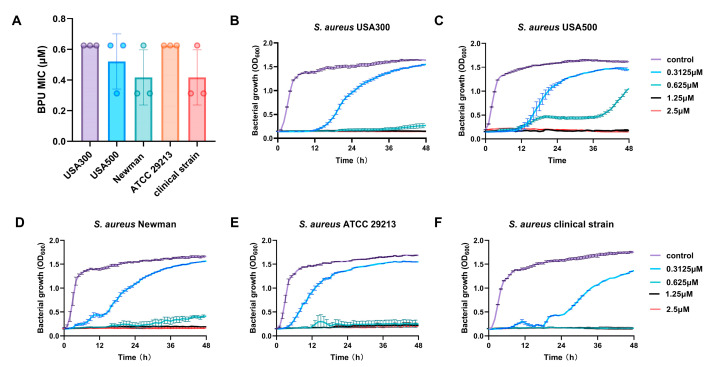
Effects of BPU on different *S. aureus* strains. (**A**) MIC values of BPU for various *S. aureus* strains, including USA300, USA500, ATCC 29213, Newman, and a clinical strain (n = 3). (**B**–**F**) Growth curves of *S. aureus* strains in response to varying concentrations of BPU.

The MIC values of BPU were measured against various *S. aureus* strains, including MRSA strains USA300 and USA500, methicillin-sensitive (MSSA) strains ATCC 29213 and Newman, and a clinically isolated strain, to assess BPU’s effect. The MIC values were consistent across these strains, ranging from approximately 0.4 to 0.6 µM, with no significant differences observed (Figure 3A). This suggests that BPU exhibits broad-spectrum activity, regardless of methicillin resistance status or clinical origin, indicating its effectiveness across diverse *S. aureus* strains, including those from oral infections. The impact of varying concentrations of BPU on the proliferation of these *S. aureus* strains was subsequently assessed (Figure 3B–F). At the highest concentration (2.5 µM), BPU completely inhibited bacterial growth across all strains. Lower concentrations (1.25 µM and 0.625 µM) resulted in varying degrees of growth inhibition and delay, while the lowest concentration (0.3125 µM) showed minimal inhibitory effects.

### 2.4. Eradication of Mature Biofilm by NaF Combined with BPU In Vitro

**Figure 4:**
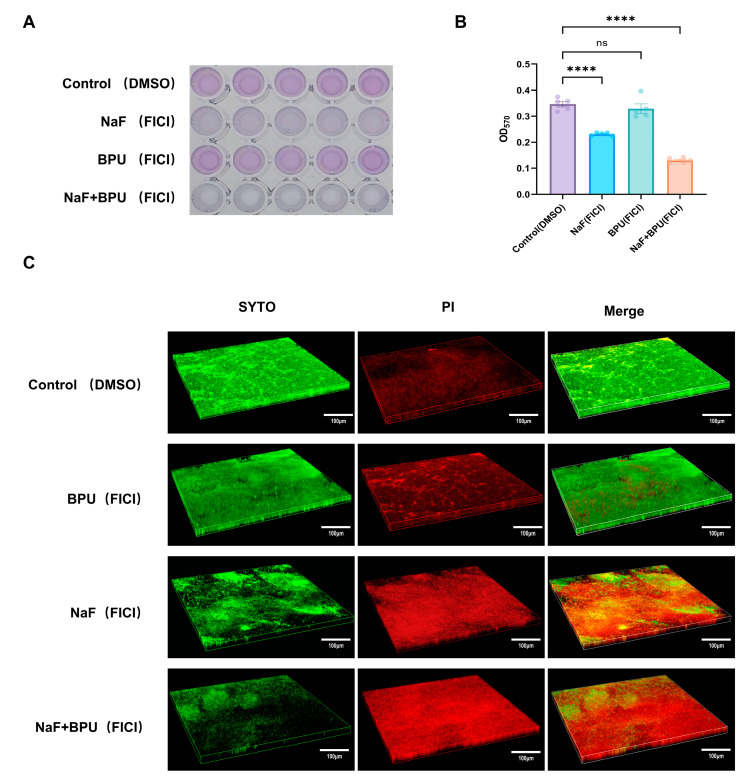
Eradication of mature biofilms by combined NaF and BPU. (**A**) Crystal violet-stained images of 24 h biofilms treated with 0.01% DMSO, NaF, BPU, or their combination. (**B**) Semi-quantitative biofilm measured by OD_570_ from five replicates (****: *p* < 0.0001, ns: not significant). (**C**) Confocal microscopy images of treated biofilms, with green (biofilm density) and red (cell damage) fluorescence quantified using ImageJ 1.54k.

Biofilms act as protective barriers, increasing microbial resistance to stress and antibiotics through their extracellular matrix and altered phenotypes [26]. To evaluate the ability of NaF, BPU, and their combination to eradicate mature biofilms, a crystal violet staining assay was performed. *S. aureus* biofilms were allowed to form in TSBG over 24 h. After removing non-adherent bacteria, 0.01% DMSO, 16 mM NaF, 156.25 nM BPU, or their combination were added to the biofilms. Figure 4A shows the results of crystal violet staining, indicating the extent of biofilm removal for each treatment. Figure 4B provides a semi-quantitative analysis, showing that the combination of NaF and BPU significantly enhanced biofilm removal compared to the control and individual treatments, achieving the greatest reduction in biofilm mass (n = 5, ****: *p* < 0.0001).

The combination group showed the lowest green fluorescence intensity (8.684), indicating the greatest decrease in biofilm density compared to NaF alone (18.83), BPU (25.171), and the control (31.354). Red fluorescence, indicating cell damage, was highest in the combination group (28.846), followed by NaF alone (25.62), BPU (15.601), and the control (10.814). These results demonstrate that the combination was significantly more effective in reducing biofilm density and increasing bacterial cell damage compared to the individual treatments (Figure 4C).

### 2.5. Homology Modeling and Molecular Docking

**Figure 5:**
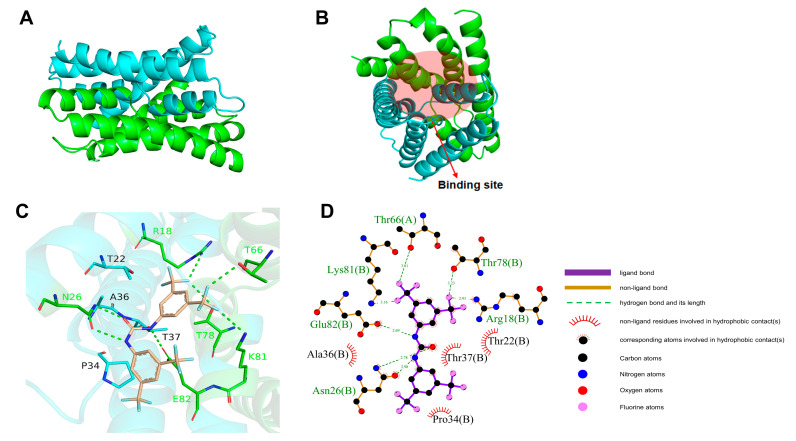
Analysis of interactions between BPU and the fluoride ion channel. (**A**) Three-dimensional structure of the dimerized protein. (**B**) Predicted binding site within the concave cavity of the dimer. (**C**) Hydrophobic interactions and hydrogen bonds between BPU and key amino acids, stabilizing the binding. (**D**) Detailed 2D interaction.

To predict the binding interactions between BPU and the fluoride ion channel protein, molecular docking was utilized. Figure 5A illustrates the three-dimensional structure of the dimerized protein. Pre-experimental predictions suggested a potential binding site for the small molecules within the concave cavity at the ends of the dimer, as depicted in Figure 5B. AutoDock 4.2.6 further confirmed the stable binding of BPU to the protein. Figure 5C,D shows the interaction analysis, revealing that BPU forms several hydrogen bonds with amino acids in both chains, as well as hydrophobic interactions that help stabilize the binding. The calculated binding energy (Table 2) was −11.528 kcal/mol, with a predicted inhibition constant (Ki) of 3.47 × 10^−9^ M, indicating a strong binding affinity between BPU and the fluoride ion channel.

### 2.6. Fluoride Ion Channel Gene Overexpression

**Figure 6:**
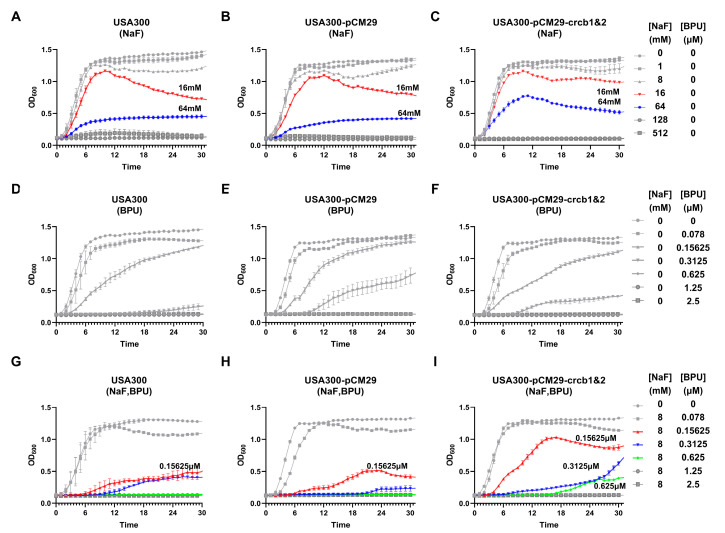
Impact of *crcB1*&*2* Overexpression on *S. aureus* USA300 Growth with NaF and BPU Treatments. (**A**–**C**) Growth of wild-type USA300 (**A**), USA300-pCM29 (**B**), and USA300-pCM29-*crcB1*&*2* (**C**) with varying NaF concentrations. *crcB1*&*2* overexpression led to higher OD_600_ values at 16 mM and 64 mM NaF. (**D**–**F**) BPU treatment showed dose-dependent inhibition across all strains, with no significant differences observed. (**G**–**I**) Combined treatment with 8 mM NaF and BPU: the strain overexpressing *crcB1*&*2* (**I**) exhibited higher OD_600_ values at 0.15625 µM, 0.3125 µM, and 0.625 µM of BPU, indicating a requirement for higher BPU concentrations to achieve similar growth inhibition.

To verify the potential targets, we cloned the fluoride ion channel genes (*crcB*1 and *crcB*2) into the pCM29 plasmid for overexpression. We then analyzed the growth curves of various *S. aureus* USA300 strains, including the wild-type strain, the strain carrying the empty pCM29 vector, and the strain carrying the pCM29-*crcB*1&2 plasmid, under different concentrations of NaF, BPU, and a combination treatment with a fixed NaF concentration (8 mM). In the wild-type USA300 and empty vector groups, growth inhibition increased dose-dependently with rising NaF concentrations. In contrast, the group overexpressing *crcB*1&2 (USA300-pCM29-*crcB*1&2) exhibited significantly higher OD_600_ values at 16 mM and 64 mM NaF, indicating increased greater tolerance to NaF compared to the other groups. Similarly, BPU treatment resulted in dose-dependent growth inhibition across all strains. Notably, the antibacterial effect of BPU remained consistent across strains, regardless of *crcB* overexpression. In the combination treatment with 8 mM NaF and BPU, significant dose-dependent growth inhibition was observed in the wild-type and empty vector groups (Figure 6G,H). However, the group overexpressing crcB1&2 (Figure 6I) exhibited higher OD_600_ values at 0.15625 µM, 0.3125 µM, and 0.625 µM of BPU, indicating that higher BPU concentrations were required to achieve similar inhibition to the other groups.

### 2.7. Evaluation of BPU Cytotoxicity

**Figure 7:**
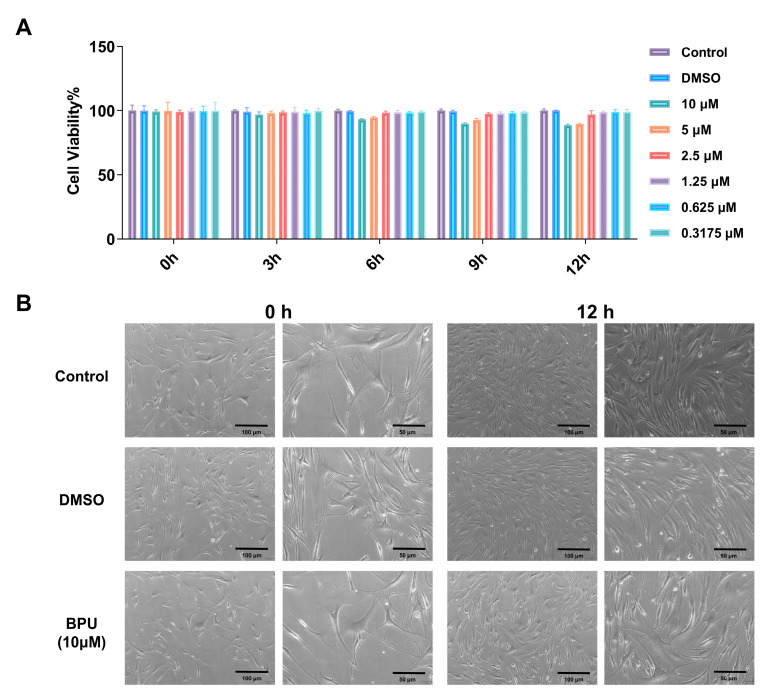
Cytotoxic Assay of BPU. The biocompatibility of BPU was assessed using HGFs. (**A**) CCK-8 assay results. (**B**) Each row corresponds to a treatment group (Control, 0.01% DMSO, and 10 µM BPU), while the two columns display the microscopic morphology of HGFs at 0 and 12 h, respectively. The images from the Control group depict the morphology of untreated cells. In each set of images, the left images are at 40× magnification with a scale bar of 100 µm, providing an overview of the cell population, while the right images are at 100× magnification with a scale bar of 50 µm, offering a closer examination of cellular morphology details.

We evaluated the cytotoxicity and proliferation of BPU on HGFs using the CCK-8 assay. At a concentration of 10 µM, BPU showed no significant cytotoxic effects on HGFs (Figure 7A). Additionally, no abnormalities were observed in cell morphology under microscopic examination, indicating that BPU did not affect the normal structure or appearance of the cells (Figure 7B).

## 3. Discussion

*S. aureus*, particularly MRSA, frequently colonizes the oral cavity, posing significant treatment challenges and public health risks [2,6,15]. While fluoride is widely used to control dental caries and promote remineralization, standard concentrations of NaF in oral care products typically range from 226 ppm to 1100 ppm (approximately 12 mM to 60 mM NaF) [27,28].

These concentrations exhibit inhibitory effects on *Porphyromonas gingivalis, Streptococcus sanguinis,* and *Streptococcus mutans* [29]. However, the impact of NaF on *S. aureus* has not been thoroughly investigated. Our study determined that the MIC of NaF against *S. aureus* strains was approximately 128 mM, which is notably higher than the fluoride concentrations found in most oral care products. This suggests that these typical fluoride levels may be insufficient to effectively inhibit *S. aureus* growth. To address this issue, we explored various compounds to enhance fluoride’s antibacterial and antibiofilm effects. Nonetheless, most of the tested compounds did not exhibit strong synergistic effects (FICI > 0.5), and some demonstrated cytotoxicity. Previous studies have demonstrated that certain small molecules, when combined with fluoride, can effectively inhibit the growth of *Streptococcus mutans* and *Escherichia coli* [30,31,32], while our experiments revealed that most of these molecules did not show significant synergistic effects against *S. aureus*. In the other compound libraries, several compounds have been identified that enhance the antibacterial effect of fluoride against *S. aureus*. However, these compounds also exhibit cytotoxic effects on human cells, which limits their potential application in clinical settings. These findings reveal the urgent need for novel strategies to combat antibiotic-resistant *S. aureus* in the oral environment.

BPU combined with NaF demonstrated strong synergistic antibacterial activity against *S. aureus* (FICI = 0.375), significantly lowering the MIC values of both agents. This FICI value aligns with other reported synergistic combinations, such as antimicrobial peptides and ethanol-extracted propolis with ibuprofen, both showing FICI values ranging from 0.187 to 0.375 [24,33]. In contrast, combinations such as vancomycin with levofloxacin (FICI = 0.75–1.25) and fosfomycin (FICI = 1.00–1.50) did not show synergy [34], underscoring the importance of FICI values in evaluating antimicrobial interactions. The FICI standard is also relevant for assessing antimicrobial combinations in oral applications. For instance, the combination of nisin with NaF against *Streptococcus mutans* showed a FICI value of 0.5, while it was 1.35 with CHX, indicating no interaction [35]. This further illustrates the utility of FICI values in evaluating antimicrobial effectiveness. In our study, the BPU–NaF combination achieved a FICI of 0.375, which is lower than that of nisin with NaF or CHX, supporting its potential in antimicrobial therapy. The unique properties of this combination in oral applications suggest the innovative potential for both its mechanism and clinical use, offering new strategies for preventing and treating oral diseases while addressing the limitations of current antibiotic therapies.

Growth curves (Figure 1C) showed substantial inhibition of *S. aureus* when 156.25 nM BPU was added to the fluoride-containing medium. The results of ion chromatography (Figure 1D) showed that when BPU and NaF were used together, the intracellular fluoride levels in the bacteria increased substantially, whereas using BPU or NaF alone did not result in a similar level of increase. This finding suggests that BPU may inhibit the efflux of fluoride, leading to its accumulation inside the cells, thereby enhancing the antibacterial activity of NaF. In contrast, although using BPU or NaF alone is effective in inhibiting bacterial growth, their individual impact on intracellular fluoride levels is limited. Therefore, the synergistic action of BPU and NaF plays a crucial role in raising the intracellular fluoride concentration, which accounts for their heightened antibacterial effect.

The time-kill assay results (Figure 2A) demonstrated the synergistic bactericidal effect of BPU and fluoride against *S. aureus*, particularly in terms of killing rate. When used alone, NaF (4 × MIC) rapidly reduced bacterial survival to nearly 0% within 0.5 h, but there was a regrowth to approximately 30% by 2 h. BPU (4 × MIC), when used alone, reduced bacterial survival to around 5% within 1 h, with no significant regrowth thereafter. However, the combination treatments at levels of 4 × FIC and 8 × FIC showed more pronounced effects. The 4 × FIC combination reduced bacterial survival to approximately 5% within 1 h with no subsequent regrowth. Notably, the 8 × FIC combination maintained bacterial survival at around 5% throughout the entire 2 h observation period, completely preventing regrowth. As demonstrated in Figure 1C, at FIC index concentrations, the combined use of NaF and BPU significantly inhibited bacterial growth, accelerating the initial killing rate and sustaining long-term inhibition. This effect likely results from their complementary mechanisms: BPU inhibits bacterial fluoride efflux, leading to the intracellular accumulation of fluoride ions. These ions inhibit key metabolic enzymes and destabilize the cell membrane, which may further increase membrane permeability, allowing more fluoride ions to enter the cytoplasm and enhance the bactericidal effect. This dual effect of rapid killing and sustained inhibition suggests that the BPU–NaF combination may offer advantages over traditional antibiotics, particularly in oral applications. From a clinical perspective, this combination shows considerable potential for treating persistent *S. aureus* infections, as it may reduce the risk of recurrence and shorten the overall treatment duration. Compared to traditional antibiotics, which may require prolonged treatment periods (such as vancomycin), the BPU–NaF combination provides a faster and more effective alternative, particularly in scenarios requiring rapid bacterial clearance.

TEM images (Figure 2) provide essential insights into the structural damage [36] caused by the combination of BPU and NaF, demonstrating its enhanced antibacterial effects against *S. aureus*. In contrast, scanning electron microscopy (SEM) focuses more on surface deformations such as cell wall changes [37]. In the control group, the intact cell wall and membrane helped maintain bacterial metabolism and stress resistance [38]. This could explain *S. aureus*’s ability to persist in the oral environment. BPU treatment alone caused minor structural changes, affecting about 20% of the cells with irregular cell walls and slight cytoplasmic alterations, consistent with moderate antibacterial effects at low concentrations [20]. NaF treatment alone led to more pronounced damage, with around 40% of cells displaying indistinct cell wall boundaries and cytoplasmic vacuoles, indicating compromised integrity. The combination of BPU and NaF induced severe ultrastructural alterations in over 80% of the cells, including extensive cell wall disruption, membrane detachment, and cytoplasmic leakage, resulting in severe membrane rupture (Figure 2K–M). About 40–50% of the cells developed vacuole-like structures, underscoring the damage from combined treatment. These changes suggest a stress response to high intracellular fluoride ion levels [39]. The observed vacuoles may represent a defense mechanism to sequester excess fluoride, attempting to maintain ionic balance [27,40]. This response intensifies with increased intracellular fluoride concentrations, leading to reduced ATP production, impaired ion pump function, and further ionic imbalance [41]. Such disruptions contribute to bacterial cell death by impairing critical cellular processes. Additional stress-induced morphological changes, such as reduced cell length and altered extracellular carbohydrate content, further weaken bacterial viability [42,43].

The consistent MIC values of BPU across various *S. aureus* strains, including MRSA, MSSA, and a clinically isolated oral strain (Figure 3), demonstrate its broad-spectrum effectiveness. This indicates that BPU’s antibacterial activity is effective against different bacterial profiles, regardless of methicillin resistance or clinical origin. More importantly, the synergistic effect between BPU and NaF suggests that this combination could be especially effective for treating oral infections, where *S. aureus* is frequently encountered. Oral infections present unique treatment challenges due to the complex environment of the oral cavity, where bacterial communities are often resistant to conventional treatments. The fact that BPU maintains low MIC values (0.4 to 0.6 µM), even against strains isolated from the oral cavity, suggests that this combination could effectively target *S. aureus* infections at lower doses, potentially minimizing side effects and reducing damage to healthy oral tissues. In dental care, where antimicrobial agents are frequently used, a treatment option that requires lower doses but remains highly effective would offer significant clinical advantages. Compared to other antimicrobials, such as vancomycin and daptomycin, which often require higher MIC values to inhibit MRSA strains, BPU consistently maintains lower MIC values across all strains tested. This indicates that BPU, when combined with NaF, could offer a safer and more effective approach to treating oral infections, reducing the risk of toxicity and minimizing the development of antibiotic resistance.

**Figure 8:**
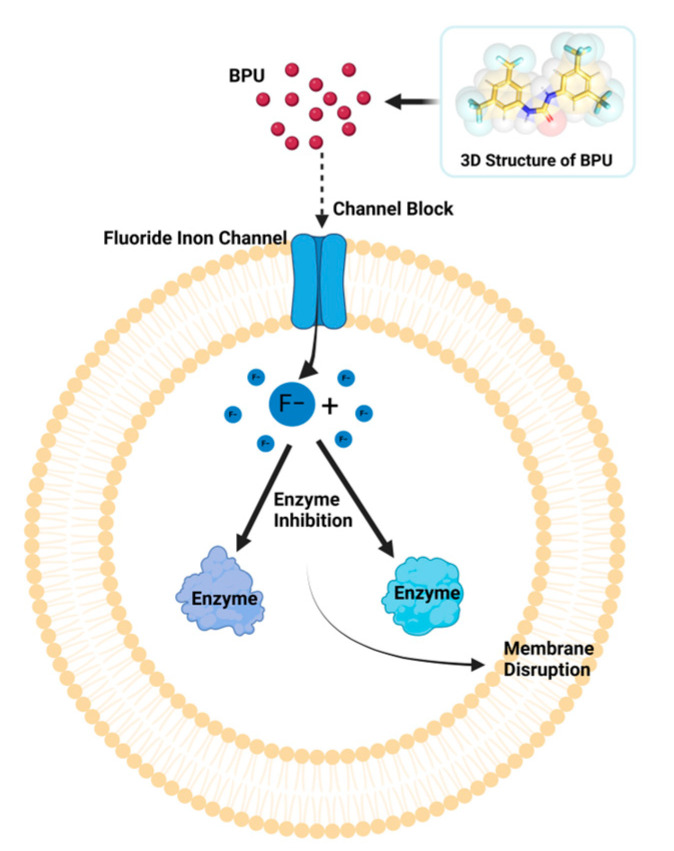
Antibacterial Mechanism of BPU and Fluoride Against *S. aureus.*

In this study, we propose that the synergistic effect of BPU with NaF is primarily due to BPU blocking bacterial fluoride ion channels, thereby preventing fluoride efflux and leading to toxic intracellular accumulation. This increased fluoride retention significantly enhances the antibacterial efficacy of NaF, as the excess intracellular fluoride disrupts key metabolic processes and induces oxidative stress, which destabilizes the bacterial cell membrane and exacerbates membrane damage. This enhanced disruption strengthens the overall antibacterial action, ultimately leading to cell death. Figure 8 illustrates the stages of this mechanism, highlighting how BPU increases intracellular fluoride levels to boost NaF toxicity, thereby enhancing its antimicrobial potency.

Biofilms persist throughout infections, making bacterial load reduction crucial for eradication. These biofilms consist of bacteria that are surrounded by a protective extracellular matrix, which includes proteins, extracellular DNA (eDNA), and polysaccharides [44]. The biofilm formed by *S. aureus* in the oral cavity exhibits unique characteristics due to its multispecies coexistence and competition. By interacting with microorganisms such as *Candida albicans, Porphyromonas gingivalis*, *Fusobacterium nucleatum*, and *Streptococcus* species, the biofilm’s structure becomes altered, thereby increasing the infection’s complexity [45,46,47,48]. This complexity, coupled with the oral environment, enhances the biofilm’s antibiotic resistance, making these infections more difficult to eradicate and more likely to become chronic. Our study demonstrates that the combination of NaF and BPU is significantly more effective against *S. aureus* biofilms compared to individual treatments. This is evidenced by a marked reduction in biofilm mass observed in the crystal violet staining assay, with the combination treatment yielding the lowest OD_570_ readings. These findings are further corroborated by confocal microscopy, which showed the lowest green fluorescence intensity in the combination treatment group, indicating a substantial decrease in biofilm density. Interestingly, NaF alone also proved to be effective in significantly reducing biofilm formation. Previous research has shown that fluoride exposure can alter gene expression in various bacteria, affecting processes related to biofilm formation, adhesion, polysaccharide production, and cell membrane structure and integrity [41,49,50]. Additionally, fluoride is known to promote cell lysis by inhibiting the glucan-binding lectin [51] within the biofilm structure and increasing peptidoglycan turnover in the cell wall [52]. In our experiments, we specifically chose sub-MIC doses of BPU and NaF to evaluate their synergistic effect in eradicating mature biofilms while minimizing potential cytotoxicity. The MIC value of NaF against *S. aureus* is 128 mM, and prolonged use of high concentrations of NaF can lead to side effects such as dental fluorosis or skeletal fluorosis. Therefore, it is essential to control NaF dosage in clinical applications. By combining BPU with NaF, we were able to reduce the NaF concentration while maintaining potent antibacterial activity. Our experimental results demonstrate that this combination significantly enhances fluoride’s regulatory effects on biofilms at sub-MIC concentrations, without the need for high doses of NaF.

Furthermore, the combination treatment led to the highest levels of bacterial cell damage, as indicated by the strongest red fluorescence intensity. This increased cell damage within the biofilm may contribute to a decrease in overall biofilm viability and resilience. The significant differences observed in biofilm reduction and cell damage between the combination treatment and the individual treatments underscore the potential of using NaF and BPU together as an effective strategy against *S. aureus* biofilms. These findings are crucial for developing new therapeutic approaches to address the inherent challenges posed by biofilm-related infections.

Traditional methods for removing *S. aureus* biofilms, such as vancomycin and mechanical clearance, have significant limitations. Clinically, vancomycin is widely used to treat *S. aureus* biofilm-associated infections; however, it requires very high concentrations (>256 µg/mL) to achieve effective biofilm eradication, which is challenging in clinical settings and poses significant toxicity risks. In dynamic environments (e.g., within the human body), even after 28 days of treatment with 2000 mg/L of vancomycin, viable bacteria can still be detected; in contrast, under static conditions, most observed bacteria appear dispersed and deformed, likely indicating cell death [53]. Additionally, mechanical removal methods often require adjunctive therapies due to the rapid regrowth of biofilms [54]. Alternative methods, such as photodynamic therapy, are effective but require specialized equipment and are cost-prohibitive. Furthermore, newer treatment modalities such as nanoparticles still require extensive safety validation [55]. CHX, a commonly used oral antimicrobial agent, can effectively reduce bacterial load in biofilms in the short term; however, its long-term use may lead to adverse effects, including tooth staining, taste alterations, mucosal irritation, and increased bacterial resistance [56]. These limitations restrict the viability of CHX as a long-term treatment option. Therefore, there is an urgent need to develop more effective and safer alternatives, such as the combination of BPU and NaF, which shows promising potential. Compared to other treatments, the combination of NaF and BPU demonstrates significant advantages in treating biofilm-associated infections in the oral cavity. NaF has been widely used in toothpastes and mouthwashes, with its safety and effectiveness in preventing cavities and controlling dental plaque being well-established. The combination of NaF and BPU not only inhibits the growth of *S. aureus* but also effectively disrupts biofilms, which is difficult to achieve with traditional antibiotics. This combination works by blocking bacterial fluoride ion channels, increasing intracellular fluoride accumulation, and inducing oxidative stress, thereby exhibiting specific effects on oral bacteria and biofilms. This presents a new potential for the treatment of localized oral infections.

Molecular docking results (Figure 5) show that BPU binds strongly to the fluoride ion channel. BPU forms seven hydrogen bonds with key amino acids: Thr66 on the A-chain, and Arg18, Asn26, Thr78, Lys81, and Glu82 on the B-chain. BPU also participates in hydrophobic interactions with four amino acids, including Ala36, which help stabilize the binding. The binding energy calculated from molecular docking is −11.528 kcal/mol, indicating a stable interaction between BPU and the fluoride ion channel. The predicted inhibition constant (*Ki*) is 3.47 × 10^−9^ M, showing a strong binding affinity. This suggests that BPU blocks the fluoride ion channel, leading to fluoride ion accumulation inside the bacterial cell. This buildup may disrupt the cell’s normal functions and enhance BPU’s antibacterial effect. Both hydrogen bonding and hydrophobic interactions contribute to the strong binding of BPU. This strong binding may prevent fluoride ions from exiting the cell, increasing fluoride toxicity and amplifying NaF’s antibacterial activity.

Our study showed that overexpression of the fluoride ion channel genes (*crcB*1 and *crcB*2) increased *S. aureus* resistance to NaF (Figure 6A–C, NaF treatment). This suggests that these genes helped remove fluoride ions from the cell, lowering their concentration inside and reducing their toxic effects. This result was consistent with previous research. Overexpression of *crcB*1 and *crcB*2 had no impact on the bacterium’s resistance to BPU (Figure 6D–F, BPU treatment), suggesting that BPU’s antimicrobial action did not directly involve these fluoride ion channels. When *S. aureus* was treated with 8 mM NaF combined with different concentrations of BPU (Figure 6G–I), strains overexpressing crcB1&2 showed higher OD_600_ values at the same BPU levels. Although the exact mechanism by which BPU interacted with fluoride ion channels remains unclear, our data suggested that BPU may have interfered with fluoride efflux. This was supported by the higher OD_600_ values observed in the crcB1&2 overexpressing strains when treated with BPU and NaF in combination. These results indicated that overexpression of crcB1 and crcB2 enhanced resistance to fluoride by actively exporting fluoride ions from the cell, thereby reducing intracellular toxicity. This resistance mechanism was likely crucial for bacterial survival in fluoride-rich environments. In contrast, BPU’s action appeared unaffected by fluoride ion channel activity alone, but when combined with NaF, it may have disrupted fluoride ion efflux, suggesting a novel approach to overcoming resistance in *S. aureus*. These findings were important for understanding how fluoride ion channels contributed to resistance mechanisms and may offer insights for developing new treatments against drug-resistant strains. Further research is needed to fully understand how BPU interacts with fluoride ion channels.

HGFs play a crucial role in maintaining gingival structure, promoting tissue repair, regulating inflammatory responses, and contributing to the progression of periodontal disease [57,58]. As a key component of gingival tissues, HGFs are highly relevant for evaluating the safety of substances that may come into contact with oral tissues, particularly in the context of antimicrobial treatments. HGFs are commonly used in cytotoxicity assays, such as the CCK-8 assay, to assess the safety of dental materials and antimicrobial agents [59,60].

In our study, we found that BPU at a concentration of 10 μM, which is much higher than the effective concentration (FICI = 0.15625 μM) against *S. aureus*, exhibited no significant cytotoxicity toward HGFs (Figure 5). Moreover, cell morphology remained normal, further confirming the non-toxic nature of BPU at this concentration. This indicates that BPU has a wide therapeutic window, as it exhibits strong antimicrobial activity against *S. aureus* at concentrations far below those that cause toxicity to human cells. The wide therapeutic window suggests that BPU could be safely used in the treatment of oral infections caused by *S. aureus*. The combination of BPU and NaF further enhances its antimicrobial effect, providing a potentially effective treatment for biofilm-associated infections in the oral cavity. Furthermore, the absence of cytotoxicity at therapeutic concentrations supports the potential clinical application of this combination, as it can effectively target biofilms without damaging host tissues. However, to fully ensure therapeutic safety, further studies, including in vivo safety assessments and longer-term exposure studies, are required to confirm the safety profile of BPU, particularly in clinical settings where repeated or prolonged exposure may occur.

Clinically, the combination of BPU and NaF presents a promising strategy for managing biofilm-associated infections in the oral cavity, such as *S. aureus*-induced oral ulcers, peri-implantitis, and osteomyelitis. Considering the limitations and side effects of current oral antiseptics such as CHX, the combination of BPU and NaF offers an effective alternative that could address these issues with enhanced antibacterial efficacy. Additionally, low-dose fluoride promotes osteogenesis, which further supports its use in treating peri-implantitis and osteomyelitis. Future studies should prioritize in vivo validation of these findings and evaluate the long-term safety and efficacy of this combination therapy in clinical settings. Moreover, exploring the molecular mechanisms underlying BPU’s enhancement of fluoride activity could provide valuable insights for designing more effective antibacterial therapies.

## 4. Materials and Methods

### 4.1. Bacterial Strains and Plasmids

In this study, various strains of *S. aureus* were utilized, including MRSA strains such as USA300, TCH1516, and USA500 2395 [61,62]. In addition, MSSA strains such as *S. aureus* Newman and ATCC 29213 were also used [63,64]. The clinical strain used in this study was provided by a dentist from the Affiliated Hospital of Yunnan University, following approval from the hospital’s Ethics Committee (Approval Number: 2024106) and the patient’s informed consent. pCM29 is a delivery vector for *Escherichia coli* and *S. aureus* [65] and was used to construct an overexpression plasmid, pCM29-*crcB*1&2.

### 4.2. Culture Media and Chemicals

*S. aureus* was grown in TSB (Oxoid, Basingstoke, UK) at a temperature of 37 °C with continuous shaking at 180 rpm. MIC tests and checkerboard microdilution assays were conducted using cation-adjusted Mueller–Hinton broth (CAMHB, Oxoid, UK) at 37 °C [66]. For the biofilm assay, *S. aureus* was cultured in TSBG (TSB supplemented with 1% glucose) at 37 °C [67,68].

Chloramphenicol (CAS No. 56-75-7; purity: 97%), ampicillin (CAS No. 69-53-4; purity: 97%), and DMSO were obtained from Sangon Biotech (Shanghai, China). NaF was sourced from Sigma-Aldrich (St. Louis, MO, USA). For strain selection, ampicillin (100 µg/mL) and chloramphenicol (10 µg/mL) were used. Lysostaphin (CAS No. 9011-93-2; Order No. A619001; Activity ≥ 1200 U/mg, BC Grade; Sangon Biotech, Shanghai, China) was used for the lysis of *S. aureus*. The enzyme was stored at 2–8 °C under dry conditions.

Several compounds were selected based on structural similarity to BPU or previous research on their antimicrobial potential and were dissolved in DMSO for the assays. The specific names, suppliers, and origins of Compounds **1**–**8** are detailed in Table 3.

### 4.3. Minimum Inhibitory Concentration

The MIC of fluoride and compounds in *S. aureus* were determined using the broth microdilution method, following the procedure described earlier [69]. As controls, we used media without antimicrobial agents as a negative control and a reference strain of *S. aureus* ATCC 29213 with known MIC values as a positive control. These controls ensured the validity of the MIC results and allowed for accurate comparisons.

### 4.4. Checkerboard Microdilution Assay for Synergistic Studies

The checkerboard method was employed to assess whether compounds act synergistically in inhibiting *S. aureus* growth with NaF. A FICI of 0.5 is defined as synergistic [70].

### 4.5. Planktonic Growth Assay

An automated growth curve detector (Bioscreen C, Turku, Finland) was used to measure the optical density at 600 nm for *S. aureus* strains. The overnight cultures were diluted at a 1:200 ratio and incubated at 37 °C with continuous shaking at 180 rpm. Measurements of the optical density at 600 nm were taken hourly.

### 4.6. Fluoride Ions in Cells

A 100 µL aliquot of an overnight culture of *S. aureus* USA300 was inoculated into 20 mL TSB in sterile 50 mL Falcon tubes. Four treatment groups were prepared: (1) 0.01% DMSO (control), (2) 8 mM NaF, (3) 56.25 nM BPU, and (4) a combination of 8 mM NaF and 156.25 nM BPU. The cultures were incubated at 37 °C with continuous shaking at 180 rpm. After 16 h of incubation, bacterial growth was measured at 600 nm (OD600). The cultures were centrifuged at 4000× *g* for 10 min at 4 °C, and the resulting pellets were washed three times with double-distilled water (ddH_2_O). The washed pellets were resuspended in 5 µg/mL lysostaphin solution and incubated at 37 °C for 30 min to lyse the cells. Lysates were filtered through a 0.22 µm syringe filter and injected into an ion chromatography system (Thermo Fisher Scientific ICS-5000+, Waltham, MA, USA). Fluoride ions were separated using an ion-exchange column, with KOH/methanesulfonic acid (30 mM) as the eluent. The flow rates for the pump and suppressor were set at 1 mL/min and 0.45 mL/min, respectively. Fluoride concentrations were detected using a conductivity detector. The samples were injected in 25 µL aliquots, and fluoride ion concentrations were quantified by comparing chromatogram peak areas with a standard curve, with results expressed in parts per million (ppm) or millimolar (mM).

### 4.7. Time-Kill Assay

The time-kill assay was conducted in accordance with standard protocols [71], with specific adaptations for our study. *S. aureus* USA300 was cultured overnight in TSB at 37 °C with continuous shaking at 180 rpm. The overnight culture was then diluted 200-fold into fresh CAMHB and incubated for an additional 2 to 3 h until it reached the logarithmic growth phase (OD600 approximately 0.4–0.6). Upon reaching the logarithmic phase, the bacterial density was adjusted to approximately 2 × 10^7^ CFU/mL. A control group was established using 0.01% DMSO. Additionally, four experimental groups were established: (1) 512 NaF, (2) 2.5 µM BPU, (3) a combination treatment using 64 mM NaF and 0.625 µM BPU at 4 × FIC concentrations, and (4) a combination treatment using 128 mM NaF and 1.25 µM BPU at 8 × FIC concentrations. Cultures were maintained at 37 °C with continuous agitation. At the time points of 0, 0.5, 1, 1.5, and 2 h, 0.1 mL samples were taken from each group, serially diluted in phosphate-buffered saline (PBS), and spread on CAMHB agar plates. The plates were incubated overnight at 37 °C, and colony-forming units (CFUs) were manually counted using a colony counter. Results were expressed in log CFU/mL, and each experiment was repeated at least three times to ensure reproducibility.

### 4.8. Transmission Electron Microscopy

After growing *S. aureus* USA300 to the logarithmic growth phase, the compounds were added to the culture. The final medium consisted of TSB containing either 0.01% DMSO (control), 156.25 nM BPU, 16 mM NaF, or a combination of 156.25 nM BPU and 16 mM NaF. The cultures were incubated at 37 °C with continuous shaking at 180 rpm for 24 h. After incubation, the cultures were centrifuged at 5000× *g* for 10 min at 4 °C to collect the cell pellets. The cell pellets were fixed in a solution of 4% paraformaldehyde and 2.5% glutaraldehyde in 0.1 M sodium cacodylate buffer (pH 7.2) for 4 h to preserve the cellular ultrastructure. The pellets were then washed three times with the same buffer and centrifuged to remove any remaining fixative. Post-fixation was performed using 2% osmium tetroxide, 0.8% potassium ferricyanide, and 5 mM calcium chloride in 0.1 M sodium cacodylate buffer for 1 h. The samples were dehydrated through a graded acetone series (30%, 50%, 70%, 90%, and 100%) and embedded in Epon 812 resin overnight. Ultrathin sections, 70–90 nm thick, were cut using an ultramicrotome, stained with uranyl acetate and lead citrate, and observed under a Hitachi TEM (Tokyo, Japan) to obtain high-resolution images of the cellular ultrastructure. The experiments were conducted in triplicate to ensure reliability and reproducibility.

### 4.9. Crystal Violet Assay

The activated *S. aureus* USA300 was inoculated at a 1:200 ratio into TSB with 1% glucose (TSBG), and 200 µL of this diluted suspension was transferred into each well of a sterile 96-well microplate. The plates were then incubated at 37 °C for 24 h to allow bacterial growth. After incubation, floating bacteria were carefully removed using a multichannel pipette, and each well was gently washed with 200 µL of PBS to remove any unattached bacteria. Subsequently, each well received 200 µL of either 0.01% DMSO, 16 mM NaF (FIC), BPU (FIC), or a combination of NaF and BPU (FIC) at their specified concentrations. The plates were incubated again at 37 °C for another 24 h. Following this second incubation, each well was rinsed in PBS three times to thoroughly eliminate unbound compounds and residual floating bacteria. Methanol (200 µL) was then added to each well to fix the biofilm for 15 min, ensuring its structural integrity. After the methanol was removed, 200 µL of 0.1% crystal violet solution was added to each well for 8 min to stain the biofilm, highlighting both the cells and the extracellular matrix. Post-staining, each well was rinsed multiple times with sterile water until the rinse was clear, ensuring excess dye was completely removed. Finally, 10% acetic acid was added to dissolve the bound crystal violet, and absorbance was measured at 570 nm using a spectrophotometer to quantify the extent of biofilm formation.

### 4.10. Three-Dimensional Biofilm Structure Using Confocal Laser Scanning Microscopy (CLSM)

Overnight cultures of *S. aureus* were diluted 1:200 in TSBG. Two milliliters of this diluted suspension were added to each glass-bottom culture dish (23 mm diameter; FluoroDish, WPI, Sarasota, FL, USA). The dishes were incubated at 37 °C for 24 h to allow bacterial growth and biofilm formation. After incubation, each dish was rinsed three times with 200 µL of PBS to remove non-adherent cells and planktonic bacteria. Then, 2 mL of a solution with 0.01% DMSO, 16 mM NaF, BPU at its FIC concentration, or a mix of NaF and BPU was added to each dish. The dishes were incubated again at 37 °C for another 24 h to support further biofilm development. After the second incubation, each dish was rinsed three times with PBS to remove remaining non-adherent cells and planktonic bacteria. The biofilms were stained with a viability kit to show live and dead cells. The kit’s instructions were followed. Dyes for marking live (SYTO9) and dead (propidium iodide, PI) cells were mixed and applied to each dish. The dishes were incubated for 15 min to ensure dye penetration. Fluorescence signals from SYTO9 (green) and PI (red) were detected with a confocal microscope set to optimal wavelengths. High-resolution cross-sectional images of each biofilm were captured through multiple scans. Three-dimensional biofilm images were generated using specialized imaging software. These images assessed the biofilm’s thickness, structural integrity, and cell viability.

### 4.11. Molecular Docking

BPU may inhibit *S. aureus* by targeting the fluoride ion channel, resulting in the accumulation of fluoride ions within the cells. To investigate this hypothesis, we constructed a dimer model of the fluoride ion channel and conducted molecular docking studies with the drug–protein complex. The dimer structure was generated using AlphaFold-Multimer (v.2.3.2), which employs deep learning algorithms to predict the three-dimensional configuration of protein dimers, thereby improving modeling accuracy. To pinpoint potential drug-binding sites, MOPAC was used to calculate surface pockets in the dimer model that could accommodate drug molecules. Molecular docking was performed with AutoDock 4.2.6, setting the docking box center at coordinates (5.637, −12.154, 5.934) to target key regions of the fluoride ion channel. The grid size was configured to 60 × 60 × 60 grid points, offering ample space to explore interactions between the drug molecules and the protein dimer. To ensure the reliability and reproducibility of the docking simulations, we carried out 50 docking runs, while other docking parameters were kept at default settings to maintain standardization. Following molecular docking, energy optimization was conducted using the Amber14 force field to precisely calculate the binding free energy of the drug–protein complex. The Amber14 force field provides high-precision molecular mechanics simulations, which help minimize the potential energy between drug molecules and the protein dimer, resulting in more stable binding conformations. For each stable docking pose, we analyzed hydrogen bonding, hydrophobic interactions, and van der Waals forces at the binding sites, allowing us to assess the binding mode of BPU within the fluoride ion channel and its potential impact on channel function.

### 4.12. Gene Overexpression in S. aureus

The *crcB1* and *crcB2* genes were amplified from the ribosome binding site (RBS) using specific primers: 5′-tgcatcggatccccgggtaccCAAACTACTTCTTTTCGAGGTGAACC-3′ (forward) and 5′-ctatgacatgattacgaattcTTAAATATGATAGCCAATATAACAAGCTATAA-3′ (reverse). This produced a 786 bp product, including the 744 bp gene sequence and plasmid homologous regions. PCR was performed using Takara high-fidelity DNA polymerase (Shiga, Japan) under the following conditions: initial denaturation at 98 °C for 10 min, followed by 30 cycles of denaturation at 98 °C for 10 s, annealing at 55 °C for 30 s, and extension at 72 °C for 2 min, with a final extension at 72 °C for 5 min. Agarose gel electrophoresis was used to confirm the product size, and the amplified fragments were extracted using the TaKaRa MiniBEST Agarose Gel DNA Extraction Kit Ver. 4.0. The purified PCR products were cloned into the pCM29 plasmid using the ClonExpress Ultra One Step Cloning Kit V2 (Vazyme Biotech, Nanjing, China). Sequence integrity was verified via colony PCR and Sanger sequencing, which was outsourced to Sangon Biotech, Shanghai. The plasmids pCM29 and pCM29-crcB1&2 were first transformed into *E. coli* DC10B and then into *S. aureus* USA300 by electroporation (2.5 kV, 200 Ω, 25 µF). Transformed *E. coli* strains were selected using ampicillin (100 µg/mL), and *S. aureus* strains using chloramphenicol (10 µg/mL).

### 4.13. In Vitro Cytotoxicity Studies

The cytotoxicity of the compounds on human gingival fibroblasts (HGFs) was assessed using the CCK-8 assay (Beyotime, Shanghai, China). HGFs were seeded at a density of 5 × 10^4^ cells/mL in 96-well plates (100 μL per well) and incubated at 37 °C with 5% CO_2_ for 24 h to allow for attachment and growth. After incubation, the supernatant was removed, and fresh DMEM containing various concentrations of BPU, 10% fetal bovine serum (FBS), and 1% penicillin/streptomycin was added to each well. Wells containing 0.01% DMSO were used as the control group. Cells were treated for 0, 3, 6, and 12 h. At each time point, 10 μL of CCK-8 reagent was added to each well, and the plates were incubated for 1 h at 37 °C with 5% CO_2_. The absorbance was measured at 450 nm using a microplate reader, and cell viability was calculated as follows:

Cell viability (%) = [(A(treated) − A(blank))/(A(control) − A(blank))] × 100%

A(treated) represents the absorbance of the treated wells, A(blank) is the absorbance of wells containing medium and CCK-8 but no cells, and A(control) is the absorbance of wells containing cells and DMSO but no BPU. Each condition was tested in triplicate, and the experiments were repeated three times to ensure reproducibility.

### 4.14. Statistical Analysis

Analysis of variance was used to compare multiple datasets, with statistical significance indicated by asterisks: ns for no significance, * for *p* < 0.05, ** for *p* < 0.01, *** for *p* < 0.001, and **** for *p* < 0.0001.

## 5. Conclusions

In summary, this study demonstrates that BPU, when combined with NaF, significantly enhances the antibacterial efficacy against *S. aureus*, particularly in challenging biofilm-associated infections. The synergistic effect of BPU is likely due to its ability to increase intracellular fluoride ion accumulation, thus amplifying the antibacterial action of fluoride. These results indicate that combining BPU with NaF could be an effective therapeutic strategy for controlling *S. aureus* infections. Future research should focus on in vivo validation and clinical trials to further explore the safety and effectiveness of this approach in treating antibiotic-resistant bacterial strains. Given the extensive use of fluoride in dental care products, the combination of fluoride with compounds shows significant potential for therapeutic applications in the field of dentistry.

## Figures and Tables

**Table 1:** MICs of NaF and various compounds against *S. aureus* USA300, with FICI values calculated to assess synergy. FICI values are interpreted as follows: ≤0.5 indicates synergy, 0.5 < FICI ≤ 4 indicates irrelevance, and >4 indicates antagonism [24,25]. Compound **1** is BPU. The specific names, suppliers, and origins of Compounds **1**–**8** are detailed in the Materials and Methods section. Other compounds with weaker synergistic effects are not listed.

Compound	Individual		Combination		FICI	Result
	NaF (mM)	Compound (nM)	NaF (mM)	Compound (nM)		
Compound **1** (BPU)	128	625	16	156.25	0.375	Synergy
Compound **2**	128	625	32	312.5	0.75	Irrelevance
Compound **3**	128	625	32	312.5	0.75	Irrelevance
Compound **4**	128	1250	32	312.5	0.5	Synergy
Compound **5**	128	2500	16	1500	0.625	Irrelevance
Compound **6**	128	100,000	64	25,000	0.75	Irrelevance
Compound **7**	128	1250	128	625	1.5	Irrelevance
Compound **8**	128	5000	64	2500	1	Irrelevance

**Table 2:** Molecular Docking Results: Binding Energy Values and Predicted *Ki*.

Compound	Binding Energy (kcal/mol)	Predicted *Ki*
BPU	−11.528	3.47 × 10^−9^ M

**Table 3:** Compounds Tested for Synergy with NaF Against *S. aureus*. The table lists compounds tested for their ability to enhance fluoride’s antimicrobial activity.

Compound Name	CAS Number	Source	Purity (%)
Compound **1** 1,3-bis [3,5-bis(trifluoromethyl)phenyl]urea, BPU	3824-74-6	Sigma-Aldrich, USA	98%
Compound **2** 1-(3-(Trifluoromethyl)phenyl)-3-(4-(trifluoromethyl)phenyl)urea	23747-75-3	ChemSrc, Shanghai, China	99%
Compound **3** 1,3-Bis(4-(trifluoromethyl)phenyl)urea	1960-88-9	ChemSrc, China	98%
Compound **4** 1-(4-Cyanophenyl)-3-(4-(trifluoromethyl)phenyl)urea	380182-97-8	ChemSrc, China	98%
Compound **5** 1,3-Bis(4-(trifluoromethyl)phenyl)thiourea	1744-07-6	ChemSrc, China	97%
Compound **6** 1-(2-Methoxyphenyl)-3-(3-trifluoromethylphenyl)urea	91286-89-4	Sigma-Aldrich, USA	99%
Compound **7** N-(3-(Trifluoromethyl)phenyl)-N’-(4-(trifluoromethyl)phenyl)urea	23747-75-3	Sigma-Aldrich, USA	98%
Compound **8** 1-(4-Isopropylphenyl)-3-(3-trifluoromethylphenyl)urea	41779-77-5	Sigma-Aldrich, USA	99%

## Data Availability

Data are contained within the article.
